# *Lst1* deficiency has a minor impact on course and outcome of the host response to influenza A H1N1 infections in mice

**DOI:** 10.1186/s12985-016-0471-0

**Published:** 2016-01-27

**Authors:** Sarah R. Leist, Heike Kollmus, Bastian Hatesuer, Ruth L. O. Lambertz, Klaus Schughart

**Affiliations:** Department of Infection Genetics, Helmholtz Centre for Infection Research, 38124 Braunschweig, Germany; University of Veterinary Medicine Hannover, Hannover, Germany; University of Tennessee Health Science Center, Memphis, Tennessee, United States of America

**Keywords:** Influenza A virus, *Lst1*, KO mouse mutant, Animal model

## Abstract

**Background:**

Previously, we performed a quantitative trait locus (QTL) mapping study in BXD recombinant inbred mice to identify host genetic factors that confer resistance to influenza A virus infection. We found *Lst1* (leukocyte specific transcript 1) as one of the most promising candidate genes in the Qivr17-2 locus because it is non-functional in DBA/2 J mice. Several studies have proposed that LST1 plays a role in the immune response to inflammatory diseases in humans and has additional immune-regulatory functions. Here, we evaluated the relevance of LST1 for the host response to influenza A infection in B6-*Lst1*^*−/−*^ mutant mice.

**Findings:**

To investigate the role of LST1, we infected B6-*Lst1*^*−/−*^ mutant and C57BL/6 N wild-type mice with a low-virulent influenza A virus (PR8M; H1N1). *Lst1* deficient mice exhibited significantly increased body weight loss at days 5 and 6 after infection and slightly increased lethality compared to infected wild-type mice. Determination of viral loads, histopathological examination and analysis of immune cell composition in bronchoalveolar lavage of infected lungs did not reveal any obvious differences between KO and wild-type mice.

**Conclusions:**

The absence of *Lst1* leads to a slightly more susceptible phenotype. However, deletion of *Lst1* in DBA/2 J mice alone does not explain the high susceptibility of this strain to PR8M influenza infections.

**Electronic supplementary material:**

The online version of this article (doi:10.1186/s12985-016-0471-0) contains supplementary material, which is available to authorized users.

## Findings

Each year, about 500 million people are infected by influenza A virus worldwide, of which about 500,000 die. We and others showed that the genetic background strongly influences the course and outcome of influenza A virus infections in different mouse inbred strains [1–4]. To identify genes that influence resistance or susceptibility to influenza A infections, we performed a genome-wide quantitative mapping study using the BXD recombinant inbred strains derived from C57BL/6 J and DBA/2 J. When infected with a low virulent isolate of PR8 influenza A virus (designated PR8M), DBA/2 J mice are highly susceptible and die within 5–7 days post infection (p.i.), whereas C57BL/6 J mice survive [1, 5]. We found about 30 candidate genes across five QTL regions [2]. One of the most promising candidates in the Qivr17-2 (quantitative trait for influenza virus resistance on chromosome 17) locus was the leukocyte specific transcript 1 (*Lst1*). The human gene *LST1* and its mouse homologue *Lst1,* formerly described as *B144* [6], are located in the MHC class III locus encoding numerous genes involved in the immune response [7, 8]. Transcripts are most abundant in immune cells, especially B cells, T cells, monocytes and dendritic cells [9]. *Lst1* expression has been shown to be up-regulated by inflammatory stimuli [8, 10]. DBA/2 J mice exhibit a deletion in the *Lst1* which results in a translational frame shift that most likely causes a premature stop codon and thus results in a truncated, non-functional protein [2]. Expression of *Lst1* transcripts was up-regulated in C57BL/6 J mice after infection with influenza A virus (PR8M; H1N1). Expression levels increased already at day 2 post infection (p.i.), showing a peak of expression at day 8 p.i.. At later time points *Lst1* expression decreased and reached levels that were similar to non-infected mice on day 18 p.i. [2, 11]. Thus, *Lst1* expression was found both during the innate and adaptive phase of the host response to influenza and peaked at the time point of T cell infiltration. Bio-GPS (http://biogps.org) expression studies show that *Lst1* was mainly expressed in immune cells including mast cells, macrophages and dendritic cells.

To further characterize the role of LST1 after influenza A infection, we studied the host response in a *Lst1* mouse knock-out (KO) model. The KO strain C57BL/6 N-*Lst1*^*tm1(KOMP)Vlcg*^ was created from the ES cell clone 12118A-B1 (obtained from the KOMP Repository; www.komp.org). It harbors a reporter-tagged deletion of the *Lst1* gene. We confirmed insertion of the targeting cassette into the coding region of exon two and three by PCR genotyping (Additional file 1: Figure S1A) and showed absence of transcripts in knock-out mice by reverse transcription PCR (Additional file 1: Figure S1B).

**Fig. 1:**
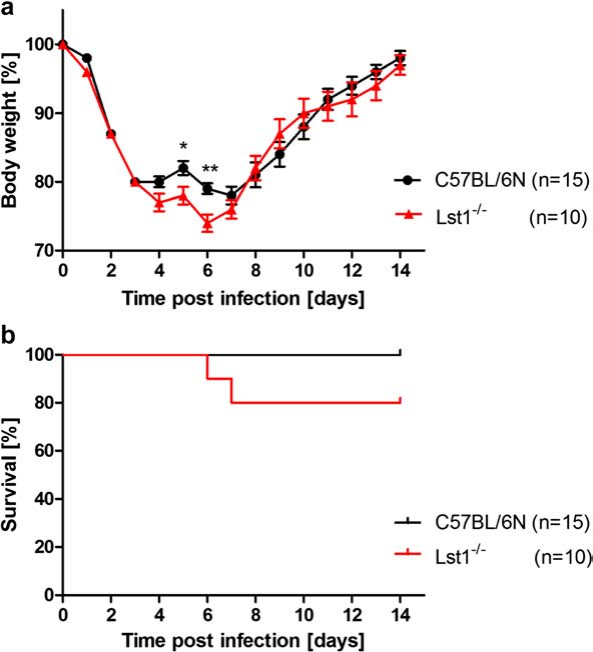
Lst1 KO mice revealed a slightly more susceptible phenotype in comparison to C57BL/6 N wild-type mice after infection with H1N1 influenza A virus. Female C57BL/6 N-*Lst1* ^*tm1(KOMP)Vlcg*^ (*n* = 10) and wild-type C57BL/6 N mice (*n* = 15) were infected intra-nasally with 2×10^5^ FFU PR8M in 20 μl PBS. Body weight (**a**) and survival (**b**) were determined for each day p.i. for a period of 14 days. Percent weight is shown with reference to the starting body weight. Significances were calculated using non-parametric Mann Whitney U test. (*p* < 0.05 for day 5; *p* < 0.01 for day 6) and Log-rank test for survival rates (not significant)

**Fig. 2:**
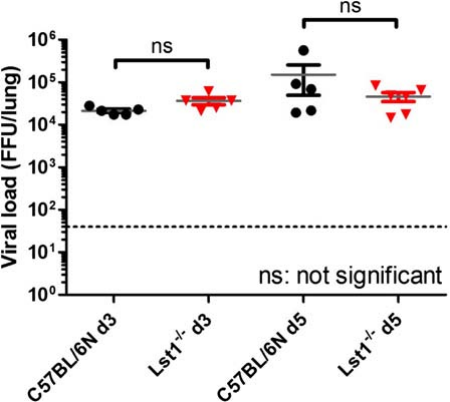
Similar viral loads in lungs of infected Lst1 KO and C57BL/6 N wild-type mice. Female C57BL/6 N-*Lst1* ^*tm1(KOMP)Vlcg*^ (*n* = 5 / time point; red) and wild type (C57BL/6 N) mice (*n* = 5 / time point; black) were infected intra-nasally with 2 × 10^5^ FFU PR8M in 20 μl PBS. Lung samples were taken on day 3 and 5 p.i.. Lungs were homogenized and viral load was titrated thrice for each sample. Each symbol represents one mouse. Mean values (*grey*) +/− SEM are depicted. Statistical analysis was performed using non-parametric Mann–Whitney U test; ns: not significant. The dotted line marks the detection limit

**Fig. 3:**
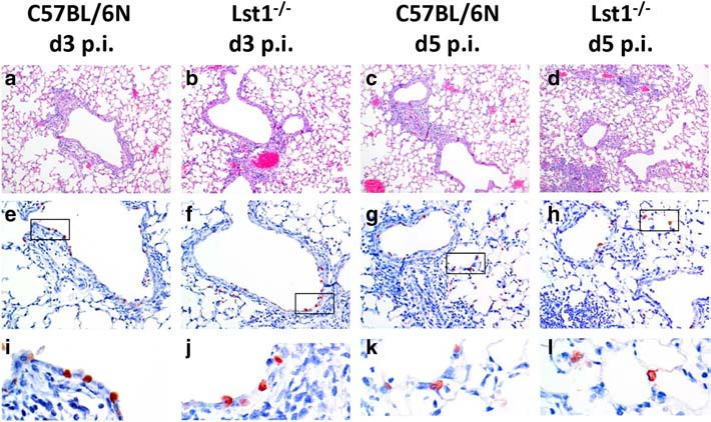
Lungs of *Lst1* KO mice and C57BL/6 N wild-type mice did not show obvious histological differences after IAV challenge. Female C57BL/6 N-*Lst1* ^*tm1(KOMP)Vlcg*^ (*n* = 3 / time point) and wild type (C57BL/6 N) mice (*n* = 3 / time point) were infected intra-nasally with 2 × 10^5^ FFU PR8M in 20 μl PBS. Lung samples were taken on day 3 and 5 p.i.. Formalin-fixed and paraffin-embedded tissue samples were sectioned at 5 μm thickness and stained with either hematoxylin and eosin or with a monoclonal antibody against the nucleoprotein of influenza A virus (anti-NP) [26]. Similar amounts of infiltrating immune cells (**a**-**d**) and similar numbers of virus-infected cells (**e**-**h**) were observed at 3 and 5 days p. i. Immuno-histochemical staining with anti-NP antibody was detected mostly in bronchiolar regions on day 3 p.i. (**i** and **j**), whereas on day 5 p.i. virus did spread further into alveolar regions in both mouse strains (**k** and **l**). A-D: Hematoxylin and eosin staining (10× magnification); e-h: anti-NP immune-histochemical staining (20× magnification); I-L: anti-NP staining (60× magnification)

**Fig. 4:**
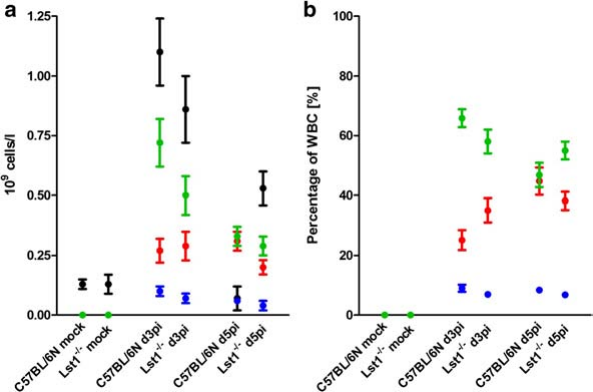
No difference in the immune cell composition in bronchoalveolar lavage in *Lst1* KO and C57BL/6 N wild-type control mice. Female C57BL/6 N-*Lst1* ^*tm1(KOMP)Vlcg*^ (*n* = 5-6 / time point) and wild type (C57BL/6 N) mice (*n* = 9-10 / time point) were infected intra-nasally with 2 × 10^5^ FFU PR8M in 20 μl PBS. On day 3 and 5 p.i. mice were euthanized by isoflurane. Bronchoalveolar lavage (BAL) was collected via instillation of 0.8 ml PBS per mouse. BAL was immediately analyzed in the hematologic system VetScan HM5 (Abaxis). Samples of mock-infected mice were collected on day 3 post PBS treatment. Absolute numbers of white blood cells (black), lymphocytes (green), granulocytes (*red*) and monocytes (*blue*) are shown in **a**. Lymphocyte, granulocyte and monocyte amounts relative to total white blood cells counts are depicted in **b**. Mean values +/− SEM are shown. Statistical analysis was performed using non-parametric Mann–Whitney U test

We then infected eight to twelve weeks old female C57BL/6 N*-Lst1*^*tm1(KOMP)Vlcg*^ as well as wild type control mice (C57BL/6 N) with 2×10^5^ focus forming units (FFU) of the low-virulent mouse-adapted A/PuertoRico/8/34 H1N1 virus (PR8M; [5]) after anesthesia with intra-peritoneal injection with a mixture of 100 mg/ml Ketamine and 20 mg/ml Xylazine. All animal experiments were approved by an external committee and according the guidelines of the animal welfare law in Germany (Permit numbers: 33.942502-04-051/09, 3392 42502-04-13/1234). Changes in body weight and survival rates were monitored for 14 days p.i. (Fig. 1). Mice with a loss of more than 30 % of the starting body weight were euthanized and recorded as dead. *Lst1* KO mice exhibited significantly increased body weight loss at days 5 and 6 p.i.. All wild-type mice survived the infection with PR8M virus whereas the KO mice showed a lethality of 20 %. However, this difference in survival was not statistical significant. No difference in survival and body weight loss was observed between wildtype and *Lst1* KO mice after infection with another influenza A virus subtype (A/Hong Kong/01/68 H3N2, [12], Additional file 1: Figure S2). Furthermore, comparison of viral loads [5] between lungs of PR8M infected *Lst1*^*−/−*^ and wild-type mice at day 3 and 5 p.i. did not reveal significant differences (Fig. 2). Histopathological analysis of infected lungs showed no obvious alteration in morphology and immune cell infiltration in KO mice compared to the wild-type controls (Fig. 3). To assess the magnitude and composition of immune cell infiltrates in the airways, we analyzed bronchoalveolar lavage (BAL). We were not able to detect significantly different amounts of immune cells neither in infected nor in mock-infected BAL fluids (Fig. 4).

In conclusion, B6-*Lst1*^*−/−*^ KO mice are slightly more susceptible to PR8M influenza A infection which is reflected in an increased body weight loss and slightly reduced survival rate. The susceptibility of KO mice is much less pronounced compared to DBA/2 J mice. Thus, our results show some contribution of Lst1 to susceptibility of DBA/2 J mice. However, its effect is rather small. Therefore, other genes with polymorphisms in Qivr17-2 as well as polymorphic genes in other QTL regions are contributing to the high susceptibility of DBA/2 J mice. Furthermore, it is conceivable that Lst1 has a major contribution by interacting with additional DBA/2 J alleles in Qivr17-2 or other QTLs that were mapped previously. These results emphasize the influence of different gene loci on the host response to influenza A H1N1 infection in mice.

There are numerous reports describing that LST1 plays an important role in the immune response to inflammatory diseases in humans [10, 13–15], in bacterial infections [10] and in signal transduction [16, 17]. It has been demonstrated that LST1 plays a crucial role for transmembrane cell to cell communication [18], which was shown to be important for the intercellular transport of bacteria and retroviruses [19]. Furthermore, the expression of the *Lst1* gene was shown to be up-regulated in response to lipopolysaccharide, interferon-γ and bacterial infections [10].

The *LST1* gene has been studied extensively at the gene and mRNA level [20], but the biological functions of the protein product are largely unknown. Overexpression of LST1 in several human cell lines leads to the formation of filopodia-like membrane protrusions [21]. Recently, it was proposed that LST1 promotes the formation of tunneling nanotubes [18]. Interestingly, it has been shown that several persistent viruses, like HIV and herpes viruses, are able to use those nanotubes for intercellular transfer [22–24]. This mechanism of viral spread has not been demonstrated for influenza A viruses. In addition, it was shown recently that tunneling nanotubes promote networking of immune cells and can mediate transfer of MHC class I molecules between distant cells [25]. This function might be disturbed in *Lst1* KO mice leading to a slightly enhanced susceptibility to PR8M influenza A. We found in other mouse knock-out lines that the low-virulent PR8 virus (PR8M, [5]) is well suitable to detect even small differences in susceptibility. However, it is still possible that *Lst1* KO mice may exhibit a larger difference in phenotype when infected with another influenza virus subtype or variant.

To our knowledge the *Lst1* knock-out mouse model described here is the first in vivo model investigating the role of LST1 during influenza A infections. However, several open questions still remain with respect to the many biological functions of LST1 in other contexts. The mouse model which we generated will help to elucidate also these other functions.

## Additional file

Additional file 1: Figure S1. Targeting and genotyping strategy of the *Lst1* KO strain. Mice were genotyped by using different primer pairs leading to PCR products which allowed the identification of wild-type (310 bp), KO (400 bp) and heterozygous animals (310 bp and 400 bp) (A). Primers (blue arrows) used for genotyping: Lst1-fwd: 5′ - GTG CGT GCT CAG TCA CAC TA – 3′; Lst1-rev: 3′ - AGG CCA ACA ATA AGT CCT TAC – 5′; neofwd: 3′ - TCA TTC TCA GTA TTG TTT TGC C – 5′. Abbreviations: *lacZ:* ß-galactosidase coding sequence from the *E.coli lacZ* gene, hubiP: promoter from the human ubiquitin C gene, *neor:* coding sequence for neomycin phosphotransferase, p(A): polyadenylation signal, black arrow: direction of gene transcription, black boxes: *Lst1* coding region. To confirm the knock-out of the *Lst1* gene in C57BL/6 N-Lst1tm1(KOMP)Vlcg mice we performed reverse transcription. **Figure S2.** No difference in changes of body weight or survival rate between *Lst1* KO and C57BL/6 J mice after infection with H3N2 influenza A virus Male C57BL/6 N-*Lst1* ^*tm1(KOMP)Vlcg*^ (*n* = 7) and C57BL/6 J mice (*n* = 10) were infected intranasally with 2x10³ FFU H3N2 virus (A/HK/01/68) in 20 μl PBS. Body weight (A) and survival (B) were determined for each day p.i. for a period of 14 days. Percent weight change is shown with reference to the starting body weight. Significances were calculated using non-parametric Mann Whitney *U* test. (*p* < 0.01 for day 1) and Logrank test for survival rates (not significant). (PDF 230 kb)
