# Supplementary material for: Lst1 deficiency has a minor impact on course and outcome of the host response to influenza A H1N1 infections in mice
Source: Virol J. 2016 Jan 27;13:17. doi: 10.1186/s12985-016-0471-0 (PMC4729168; doi:10.1186/s12985-016-0471-0)
Supplement: Additional file 1: Figure S1. — Targeting and genotyping strategy of the Lst1 KO strain. Mice were genotyped by using different primer pairs leading to PCR products which allowed the identification of wild-type (310 bp), KO (400 bp) and heterozygous animals (310 bp and 400 bp) (A). Primers (blue arrows) used for genotyping: Lst1-fwd: 5′ - GTG CGT GCT CAG TCA CAC TA – 3′; Lst1-rev: 3′ - AGG CCA ACA ATA AGT CCT TAC – 5′; neofwd: 3′ - TCA TTC TCA GTA TTG TTT TGC C – 5′. Abbreviations: lacZ: ß-galactosidase coding sequence from the E.coli lacZ gene, hubiP: promoter from the human ubiquitin C gene, neor: coding sequence for neomycin phosphotransferase, p(A): polyadenylation signal, black arrow: direction of gene transcription, black boxes: Lst1 coding region. To confirm the knock-out of the Lst1 gene in C57BL/6 N-Lst1tm1(KOMP)Vlcg mice we performed reverse transcription. Figure S2. No difference in changes of body weight or survival rate between Lst1 KO and C57BL/6 J mice after infection with H3N2 influenza A virus Male C57BL/6 N-Lst1 tm1(KOMP)Vlcg (n = 7) and C57BL/6 J mice (n = 10) were infected intranasally with 2x10³ FFU H3N2 virus (A/HK/01/68) in 20 μl PBS. Body weight (A) and survival (B) were determined for each day p.i. for a period of 14 days. Percent weight change is shown with reference to the starting body weight. Significances were calculated using non-parametric Mann Whitney U test. (p < 0.01 for day 1) and Logrank test for survival rates (not significant). (PDF 230 kb) [file 12985_2016_471_MOESM1_ESM.pdf]

Supplements

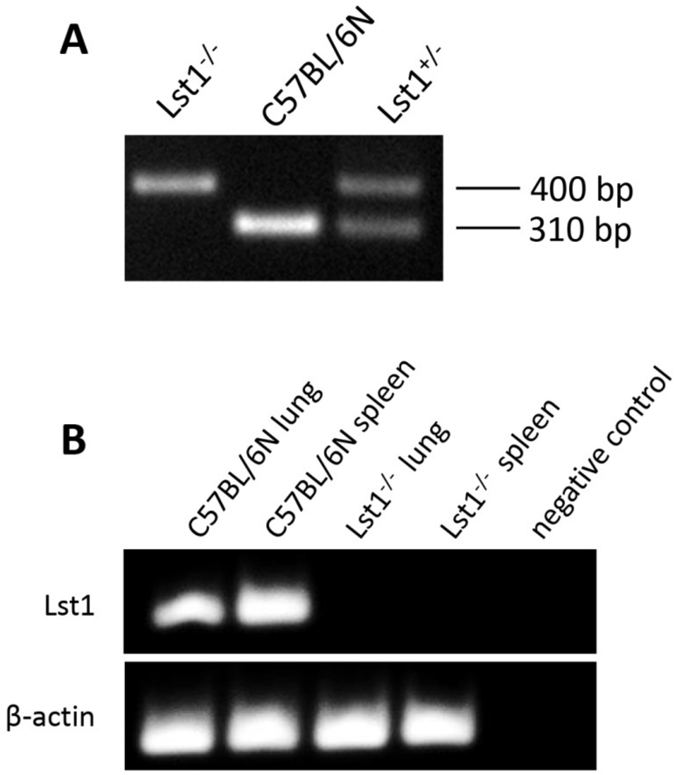

Additional Figure S1 *Targeting and genotyping strategy of the Lst1 KO strain.*

Mice were genotyped by using different primer pairs leading to PCR products which allowed the identification of wild-type (310 bp), KO (400 bp) and heterozygous animals (310 bp and 400 bp) (**A**). Primers (blue arrows) used for genotyping: Lst1-fwd: 5' - GTG CGT GCT CAG TCA CAC TA – 3'; Lst1-rev: 3' - AGG CCA ACA ATA AGT CCT TAC – 5'; neofwd: 3' - TCA TTC TCA GTA TTG TTT TGC C – 5'. Abbreviations: *lacZ:* ß-galactosidase coding sequence from the *E.coli lacZ* gene, hubiP: promoter from the human ubiquitin C gene, *neo^{r}:* coding sequence for neomycin phosphotransferase, p(A): polyadenylation signal, black arrow: direction of gene transcription, black boxes: *Lst1* coding region. To confirm the knock-out of the *Lst1* gene in C57BL/6N-Lst1$^{tm1(KOMP)Vlcg}$ mice we performed reverse transcription

PCR. For this, lungs and spleens of non-infected *Lst1* KO and wild-type mice were prepared, washed in PBS and stored in 2 ml RNAlater (Qiagen). Lungs were homogenized using the PolyTron 2100 homogenizer and total RNA was prepared using the RNaesy Midi Kit (Qiagen). 200ng of total RNA was reverse transcribed into cDNA using SuperScript III reverse transcriptase (Invitrogen TM, USA). 0.5µl of cDNA product were amplified with specific primers (LST1-F: 5'- CCT GCT TGT CAT CAT CCT GTT CAT CTG C -3'; LST1-R: 5'- TCA AGT GGG TGT GCT CCT GGC GAT G-3') to determine expression of *Lst1*. C57BL/6N wild-type mice yielded a 202bp product whereas this product was absent in Lst1 KO mice (**B**). As control, the amplification of a product specific to ß-actin is shown in both wild type and KO samples.

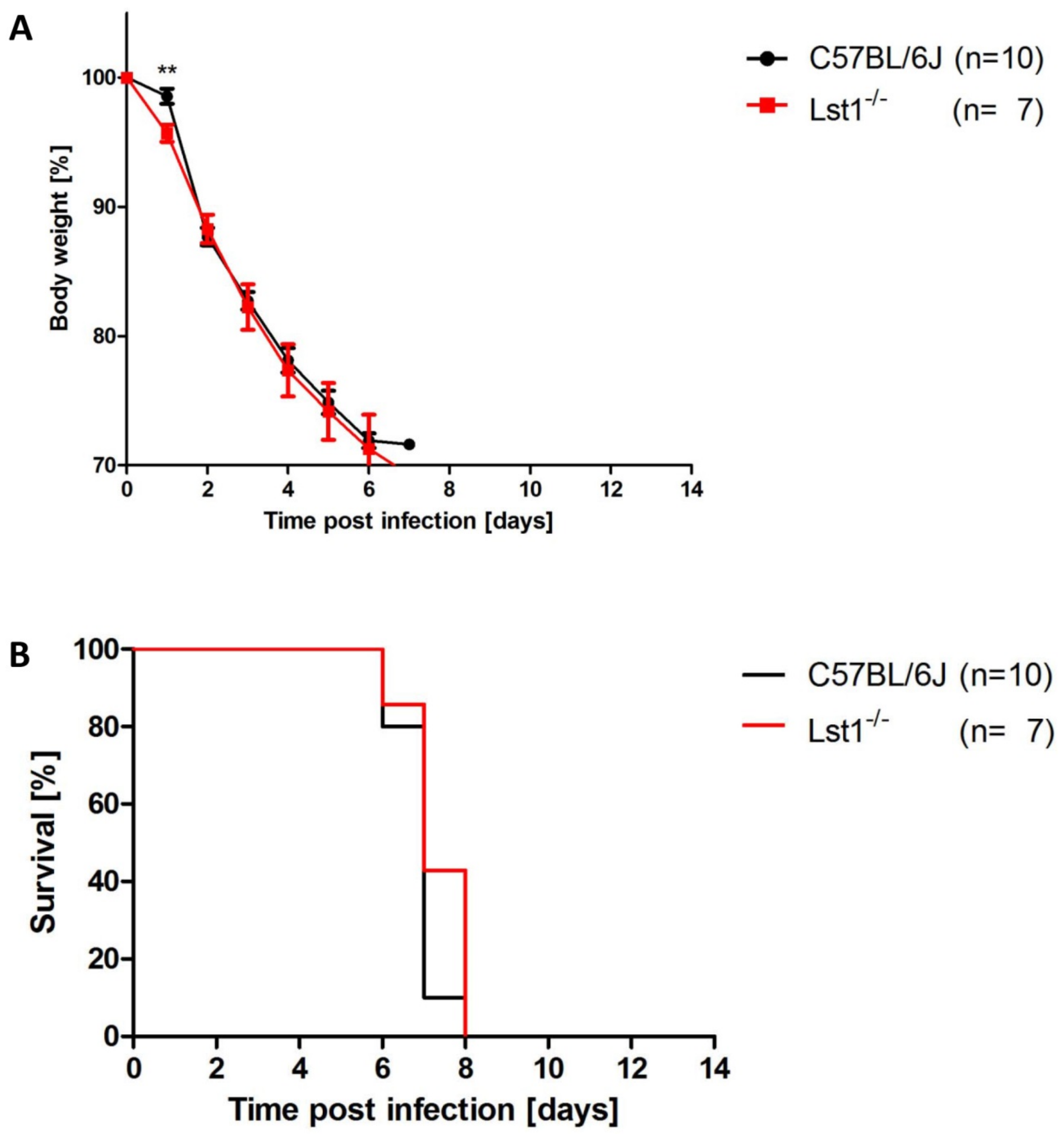

Additional Figure S2 *No difference in changes of body weight or survival rate between Lst1 KO and C57BL/6J mice after infection with H3N2 influenza A virus*

Male C57BL/6N-Lst1t$^{m1(KOMP)Vlcg}$ (n=7) and C57BL/6J mice (n=10) were infected intra-nasally with 2x10^3 FFU H3N2 virus (A/HK/01/68) in 20µl PBS. Body weight (A) and survival (B) were determined for each day p.i. for a period of 14 days. Percent weight change is shown with reference to the starting body weight. Significances were calculated using non-parametric Mann Whitney U test. ($p<0.01$ for day 1) and Log-rank test for survival rates (not significant).
